# Mesenchymal Cell Reprogramming in Experimental *MPL^W515L^* Mouse Model of Myelofibrosis

**DOI:** 10.1371/journal.pone.0166014

**Published:** 2017-01-30

**Authors:** Ying Han, Lanzhu Yue, Max Wei, Xiubao Ren, Zonghong Shao, Ling Zhang, Ross L. Levine, Pearlie K. Epling-Burnette

**Affiliations:** 1 Department of Immunology, Moffitt Cancer Center, Tampa, Florida, United States of America; 2 Department of Biotherapy, Tianjin Medical University Cancer Institute and Hospital, National Clinical Research Center of Cancer, Key Laboratory of Cancer Immunology and Biotherapy, Key Laboratory of Cancer Prevention and Therapy, Tianjin, PR China; 3 Department of Hematology, Tianjin medical University General Hospital, Tianjin, PR China; 4 Department of Hematopathology, Moffitt Cancer Center, Tampa, Florida, United States of America; 5 Leukemia Center, Memorial Sloan Kettering Cancer Center, New York City, New York, United States of America; B.C. Cancer Agency, CANADA

## Abstract

Myelofibrosis is an indicator of poor prognosis in myeloproliferative neoplasms (MPNs), but the precise mechanism(s) contributing to extracellular matrix remodeling and collagen deposition in the bone marrow (BM) niche remains unanswered. In this study, we isolated mesenchymal stromal cells (MSCs) from mice transplanted with wild-type thrombopoietin receptor (*MPL*^*WT*^) and *MPL*^*W515L*^ retroviral-transduced bone marrow. Using MSCs derived from *MPL*^*W515*^-transplant recipients, excessive collagen deposition was maintained in the absence of the virus and neoplastic hematopoietic cells suggested that the MSCs were reprogrammed *in vivo*. TGFβ production by malignant megakaryocytes plays a definitive role promoting myelofibrosis in MPNs. However, TGFβ was equally expressed by MSCs derived from *MPL*^*WT*^ and *MPL*^*W515L*^ expressing mice and the addition of neutralizing anti-TGFβ antibody only partially reduced collagen secretion *in vitro*. Interestingly, profibrotic MSCs displayed increased levels of pSmad3 and pSTAT3 suggesting that inflammatory mediators cooperating with the TGFβ-receptor signaling may maintain the aberrant phenotype *ex vivo*. FGFb is a known suppressor of TGFβ signaling. Reduced collagen deposition by FGFb-treated MSCs derived from *MPL*^*W515L*^ mice suggests that the activating pathway is vulnerable to this suppressive mediator. Therefore, our findings have implications for the future investigation of therapies to reverse fibrosis in MPNs.

## Introduction

Polycythemia vera (PV), essential thrombocytosis (ET) and primary myelofibrosis (PMF) are classified by the World Health Organization (WHO) as BCR-ABL negative myeloproliferative neoplasms (MPNs) (S1 Fig)[1]. All of these patients are at high risk for the development of myelofibrosis and acute myeloid leukemia. Although *JAK2*^*V617F*^ is a defining mutation among MPN patients[2], the majority of *JAK2*^*V617F*^-negative MPN patients have JAK-STAT activation caused by a mutation in calreticulin (*CALR*)[3–6] or the thrombopoietin receptor (*MPL*)[2]. Conclusive association between these genetic variants and myeloproliferation has been demonstrated through animal models[7–9]. However, it is currently unclear how these genetic events induce fibrosis. The presence of malignant hematopoietic cells (HPCs) are thought to be causative, albeit indirectly, in the fibrotic phenotype due to the release of pro-fibrotic factors such as TGFβ that phenotypically alter the mesenchymal compartment in the bone marrow niche[10, 11].

A deeper understanding of the biological processes underlying the development of myelofibrosis is necessary to modify or reverse this process. However, fibrosis is certainly not unique to PMF or fibrosis secondary to MPNs. Understanding uniform mechanisms contributing to the development of myelofibrosis among different types of hematologic diseases may provide insight into the cause of this phenotypic change. Moreover, the goal is to develop new avenues of therapy that can be applied across different oncologic sub-entities to prevent or reverse the aberrant process. Here, we show the phenotypic and cellular changes in MSCs derived from a mouse model of MPN and define signaling vulnerabilities that may be exploited to reverse the fibrotic phenotype.

## Material and Methods

### Murine model of *MPL*^*W515L*^-associated bone marrow fibrosis

#### Bone marrow transplantation

Retroviral vectors and a MPN model was created, as previously described (S1A Fig)[8]. In brief, female C57BL/6j donor mice were purchased from Jackson Laboratory (Bar Harbor, ME; USA). Using mice from 6- to 9 weeks of age, a single dose of 5-fluorouracil (5-FU) treatment (150 mg/kg mouse in PBS) was administered to increase progenitor cell pools 10 days prior to bone marrow transplantation (BMT). Two days prior to transplantation, bone marrow cells were isolated from donor mice for viral infection. These donor cells were then infected with recombinant retroviruses using a spinfection approach. Four million cells were prepared for each treatment group, 1 ml of viral supernatant was added to 30 μl 1M HEPES buffer (Gibco) and 2 μl polybrene and the solution was then subjected to 2500rpm for 90 minutes at 30°C. Thereafter, the cells were resuspended in 3 ml of transplant media containing 6 ng/ml recombinant murine IL-3 (R&D Systems), 10 ng/ml murine SCF (R&D Systems), and 10 ng/ml murine IL-6 for 24 hours at 37°C and 5% CO2. Viral constructs used in this project included MSCV-human-*MPL*^W515L^-GFP, MSCV-human-*MPL*^WT^-GFP or MSCV-GFP control. The spinfection procedure followed by 24 hour culture period was repeated twice to achieve optimal virus infection (S2B Fig). Recipient mice were prepared with lethal irradiation (450 cGy per day on day -2 and day -1 before transplantation) prior to tail vein injection of donor marrow cells on day 0. The experimental model was confirmed in both C57BL/6j and BALB/cj mice (data not shown). After the indicated number of days post-transplantation, CBCs and histopathology of the recipient mice were performed. Using both tibia and femurs, MSCs cultures were established. For chimeric BMT, B6.SJL-*Ptprca* (CD45.1+) BM cells were infected with *MPL*^W515L^-GFP virus and mixed at defined ratios with C57BL/6j-*Ptprcb* (CD45.2+) BM infected with *MPL*^*WT*^–GFP virus prior to injection into lethally irradiated CD45.2+ recipient mice. All mice were maintained in a pathogen-free facility at Moffitt Cancer Center. Research using animals was conducted according to international guidelines and approved by the University of South Florida Animal Care and Use Committee (IACUC). The protocol was reviewed and approved and all ethical considerations were applied. Euthanasia by inhalation of carbon dioxide from a compressed tank source was used with exposure to increasing concentrations of CO2 (displacement rate from 10% to 30% of the chamber volume/min) to avoid or minimize discomfort or distress. The CO2 fill rate did not exceed 30% of the chamber volume per minute according to IACUC approved procedures. This procedure was followed by the assurance of the cessation of cardiovascular and respiratory movements by prolonged observation at room air for longer than 10 min, or by employing a secondary method of euthanasia such as cervical dislocation, decapitation, or bilateral thoracotomy. Recipients of adoptively transferred cells received total body irradiation, immediately followed by bone marrow engraftment. Although excess pain or distress was not anticipated, radiation sickness remained a possibility. Although no adverse events were noted such as irritability, watering of the eyes, lethargy, and diarrhea, the protocol mandated that the mice would be humanely and immediately euthanized and no pain or stress relieving agents administered. Additional signs of distress that would have warrant euthanasia include shallow, rapid/or labored breathing, hypoactivity, hyperactivity, restlessness, self-trauma, aggressiveness, isolation from cage mates, ataxia, pale mucous membranes, cyanosis, failure to groom, soiled anogenital area, inactivity, failure to respond to stimuli, ruffled hair coat, piloerection, matted hair coat, lack of inquisitiveness, vocalization, and/or hunched posture. The total number of 66 mice were used for this study including both donor and transplantation recipients.

#### Virus production

293T cells were used for virus production. One day before transfection, 293T cells (2.6 × 106) were plated in a 10cm dish in DMEM medium supplemented with 25mM HEPES. The cells were then transfected with MSCV-human-*MPL*^W515L^-GFP or MSCV-human-*MPL*^WT^-GFP plasmids together with PVPack-gag/pol and PVPack-Eco plasmids and 2.5M CaCl2. Viral supernatant was collected 48 hours after transfection and used immediately (ie, fresh virus) or after freezing at -80°C. Virus used immediately generated higher peripheral blood counts on day 17 compared to frozen viral stocks. Frozen stocks were used exclusively for the chimeric transplantation model.

#### Mouse pathology

Bone marrows, spleen, and liver were obtained for pathological evaluation 17 days after transplantation of infected bone marrow cells. Wright-Giemsa stain was used to examine hematopoietic cells and whole femurs were decalcified in nitrical (5% nitric acid), and embedded in paraffin. Sections (2–4 μm) were mounted for hematoxylin and eosin (H&E) staining, Gomori’s silver impregnation (reticulin), and Masson-Medical Chemical Corporation trichrome staining. Myelofibrosis in the bone marrow was scored based on reticulin staining using the European consensus grading scale[12].

#### Primary MSC cultures from human and murine bone marrow

Primary MSCs were established, as described previously[13, 14]. Bone marrow aspirates from healthy human volunteers were obtained commercially (Lonza, Walkersville, MD). Continuous growth was carried out for human and murine MSCs in separate closed incubator chambers separate closed incubator chambers containing 5% CO2, 93% nitrogen and 2% O2 at 37° in media containing αMEM (Life Technologies Invitrogen, San Diego, CA) supplemented with 10% fetal bovine serum (Life Technologies Invitrogen), streptomycin (100 U/ml) and penicillin (100 U/ml). Differentiation of murine MSCs into adipocytes was conducted with adipogenic differentiation media for human and mouse mesenchymal stem cells (StemCell Technologies, Vancouver, Canada) and chondrocytes using chondrogenic differentiation media (R&D Systems, Minneapolis, MN). Differentiation was performed and tested using similar methods described previously for human cells[13]. Differentiation was carried out for 14–21 days in culture based on the cell lineage.

### Immunofluorescence staining of collagen matrix

To stain collagen fibers deposited into three-dimensional complexes, MSCs were grown on Lab-Tek chamber slides (Nalgene Nunc, Rochester, NY) and native collagen was allowed to form fibrillar structures. Polychromatic immunofluorescence collagen stains were performed for collagen types I (Abcam, Cambridge, MA, cat#ab6308), III (Abcam, cat#ab7778), and IV (EMD Millipore, Billerica, MA, cat#AB8201) after first screening for major subtypes (data not shown), as described previously[13]. Secondary stains were used to visualize the complexes including DyLight549 F(ab’)2 donkey anti-mouse, DyLight647 F(ab’)2 donkey anti-goat, and DyLight488 F(ab’)2 donkey anti-rabbit (Jackson ImmunoResearch laboratories, West Grove, PA) following visualization of the nuclei with DAPI (Vectashield, Vector laboratories, Burlingame, CA). Immunofluorescence was performed in a light-protected environment to preserve the intensity of staining. To visualize the fluorescence, a Leica DM16000 inverted microscope, TSC SP5 confocal scanner and a 20X/0.7NA Plan Apochromat oil immersion objective (Leica Microsystems, Wetzlar, Germany) was used. The 405 Diode, Argon, HeNe 543, NeNe 594, and HeNe 633 lasers were applied to minimize channel interference, as described previously[13]. Images were analyzed for mean pixel intensity for each field using the LAS AF lite version 2.6 software system (Leica Microsystems CMS, Wetzlar, Germany).

### Western blot analysis

MSCs were lysed with RIPA buffer (Sigma-Aldrich, St. Louis, MO) containing protease inhibitors (Roche, Basel, Switzerland) and phosphatase inhibitor cocktails (Sigma-Aldrich, St. Louis, MO). Protein concentrations were determined with Pierce BCA protein assay kit (Thermo Fisher Scientific, Waltham, MA). Proteins were separated on a 4–12% Bis-Tris gradient electrophoresis gel (Life Technologies, Carlsbad, CA) and transferred onto a nitrocellulose membrane. The membrane was then blocked in either 5% nonfat dry milk or 5% BSA followed by incubation with primary antibodies at 4°C overnight. Antibodies for pSmad3 (Abcam ab52903), Smad3 (Abcam ab40854), p-STAT3 (Cell Signaling 9145) and STAT3 (Cell Signaling 9139) were used. β–actin was used as loading control. Bands were quantified using Image J software (version 1.49).

### ELISA assay

To determine TGFβ concentration in MSC cultures, supernatants were collected and analyzed using an ELISA kit (R&D Systems, Minneapolis, MN) following manufacturer’s instructions.

### qRT-PCR for collagen

Quantitative real-time-PCR was used to investigate collagen I (*Col1A1*), III (*Col3A1*), and IV (*Col4A1*) mRNA expression in human and mouse MSCs following total RNA extraction. Total RNA was extracted from cultured MSCs using the RNeasy Micro Kit (Qiagen, Chatsworth, CA). Reverse transcription was performed using the high-capacity cDNA reverse transcription kit (Bio-Rad laboratories, Hercules, CA) in accordance with the manufacturer’s suggestions. Relative collagen gene expression was relative to TATA-binding protein (TBP), which was used as a reference gene. All primers for qRT-PCR were obtained from Life Technologies, Carlsbad, CA. The reaction mixture consisted of 1X Taqman mixture, 1X primer of each collagen or TBP. All samples for both the collagen and TBP were tested in triplicate in the same plate. The expression level of each collagen in the sample was calculated with the ΔΔCt method[15].

### Statistical analysis

Statistical significance was calculated by GraphPad Prism software v5.03 (GraphPad Software, La Jolla, Ca). Analysis of Variance (ANOVA) was used to determine the differences between the means of independent groups. Comparisons between groups were made using two-tailed t test or using nonparametric test.

## Results and Discussion

### Murine model of *MPL*^*W515L*^-associated myeloproliferation

**Fig 1:**
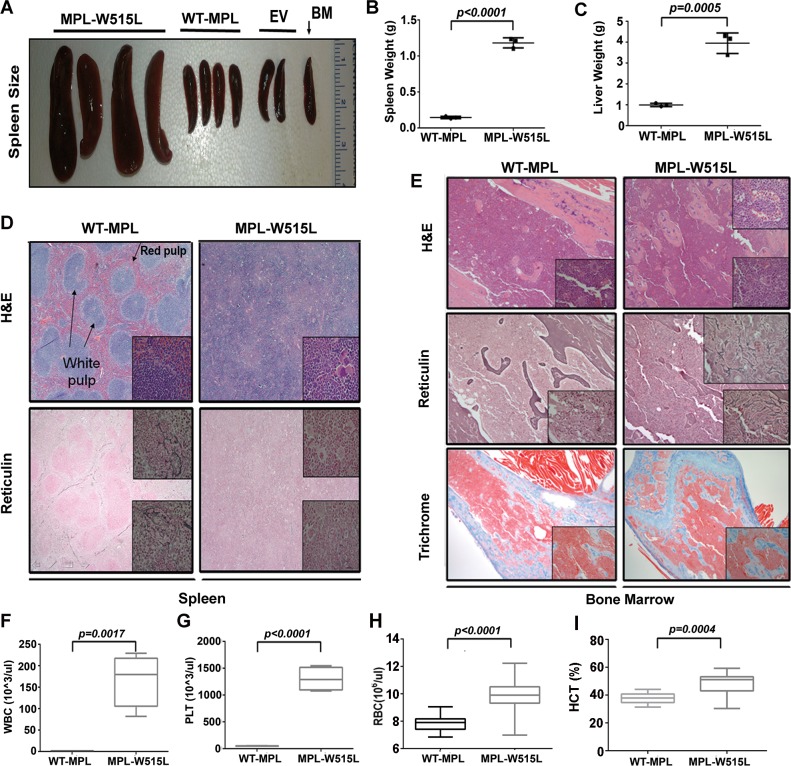
Transplantation model of *MPL*^W515L^ MPN. C57BL/6j bone marrow transplantation recipients received *MPL*^W515L^, *MPL*^*WT*^, and empty vector control (EV) infected bone marrow (BM) for 17 days. (A) spleen size (B) spleen weight (g), (C) liver weight (g), (D) H&E and reticulin stained spleens (E) H&E, reticulin, and trichrome stained bone marrow sections, (F) WBC counts (G) platelet counts (H) RBC counts and (I) hematocrit (Hct) levels were determined on day 17. Data using four mice in one representative experiment out of three replicates with statistical analysis using two-tailed t test. Significant p-values <0.05 are shown.

**Fig 2:**
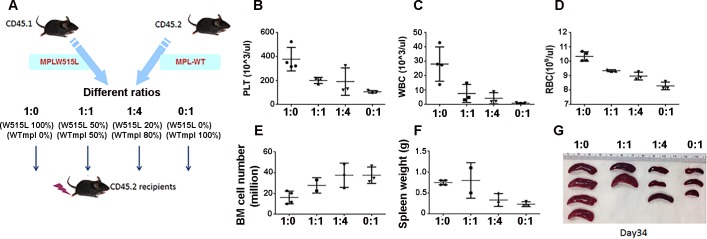
Chimeric transplantations. (A) Provides a diagram outlining the procedures in this experiment. BM from B6.SJL-*Ptprc*^*a*^ (CD45.1+) mice was infected with *MPL*^W515L^-GFP virus and mixed 1:0 (100%, n = 4), 1:1 (50%, n = 3 with one death on day 17), 1:4 (20%, n = 3) and 0:1 (0%, n = 3) with BM from B6.SJL-*Ptprc*^*a*^ (CD45.1+) mice infected with *MPL*^*WT*^ *virus*. Populations were injected into lethally irradiated C57BL/6j-*Ptprc*^*b*^ (CD45.2+) recipient mice and then (B, E) platelet count, (C, F) WBC count (D, G) RBC count were determined on day 17 (B, C, D) or day 34 (E, F, G) post-transplantation. On day 34, all mice were euthanized and (H) total BM cell number from two femurs and two tibia (I) spleen weight, and (J) pictures of spleens were determined. This is characteristic of two additional experiments. Data is shown for one representative experiment out of three with statistical analysis conducted using ANOVA. Significant p-values <0.05 are shown.

To establish an animal model of myelofibrosis, *MPL*^W515L^-virsus transduced bone marrow was injected into wild-type (WT) C57BL/6j recipients and results were compared to mice transplanted with MSCV-IRES-EGFP vector and *MPL*^WT^-viral-transduced BM (S2 Fig) using constructs and methods that were described previously[8]. In recipient mice, the animals were given lethal irradiation prior to transplantation and complete blood counts and histopathological examination of the bone marrow was carried out at various times post-transplantation to determine the optimal point of disease initiation. As shown in Fig 1A, *MPL*^W515L^-, but not *MPL*^*WT*^ or empty vector transplant recipients developed significant spleen enlargement indicated by greater spleen weight (Fig 1B) as well as increased liver weight (Fig 1C) on day 17 after transplantation. The extent of myeloproliferation was evident by ablation of normal splenic architecture including the obliteration of the red and white pulp regions (Fig 1D) and the presence of prominent atypical megakaryocytes: some with hyperlobation and disjointed nuclei, and extramedullary hematopoiesis including erythroid islands and myeloid precursors with left shift maturation in the spleen. No excess blasts were observed. Spleen (Fig 1D) and liver (not shown) reticulin stains failed to show an increase in collagen fibers with *MPL*^W515L^ retrovirus indicating that the primary location of fibrosis is in the bone marrow. As shown in Fig 1E, microscopic examination of stained BM sections revealed >95% cellularity with numerous normal appearing megakaryocytes in *MPL*^WT^ recipients. While examination of the bone marrow from *MPL*^W515L^ expressing bone marrow showed similar cellularity, focally atypical megakaryocytes with hyperchromatic nuclei were identified. Additional abnormalities included slightly expanded focal sinusoidal spaces that are filled with mature and immature hematopoietic elements characteristic of intrasinasoidal hematopoiesis. This feature is commonly present in human marrow from patients with primary myelofibrosis. Reticulin stains (detecting primarily collagen III) performed on the bone marrow of mice receiving transplants with *MPL*^*WT*^ showed no increase in reticulin fibers (overall score 0 of 3) according to the European Consensus scoring system for myelofibrosis. Reticulin fibrosis was present with a European consensus fibrosis score of 2 to 3 of 3 in the bone marrow and particularly in subcortical bone marrow spaces (Fig 1E) in all mice transplanted with *MPL*^*W515L*^-transduced cells. Trichrome stains (collagen I) were unremarkable at 17 days for both the *MPL*^*WT*^ and *MPL*^W515L^ transplant recipients, however, thickened bony trabeculae in the subcortical region of the bone was induced by *MPL*^*W515L*^ consistent with human PMF. In the peripheral blood, *MPL*^W515L^-induced leukocytosis, thrombocytosis, elevated red blood cells (RBC) and hematocrit (HCT) (Fig 1F–1H, respectively) were characteristic of this mouse model created in Balb/c mice[8] and demonstrated the effect of *MPL*^W515L^ on hematopoiesis. To further evaluate the engraftment efficacy in blood and effects on spleen size, competitive bone marrow transplants were conducted with defined ratios of *MPL*^*WT*^ and *MPL*^*W515L*^ transduced bone marrow (Fig 2A). The platelet counts (Fig 2B and 2E), WBC counts (Fig 2C and 2F), spleen size and weight were positively correlated with the number of *MPL*^*W515L*^ transplanted bone marrow (Fig 2F and 2G). The RBC count (Fig 2D) was also positively correlated with cell dose on day 17, but equalized by day 34 suggesting that normal erythrocyte differentiation requires a longer period for full reconstitution. Interestingly, a dose dependent decrease in total hematopoietic cells occurred in the bone marrow based on the dose of *MPL*^*W515L*^ (Fig 2E) expressing cells. This paradoxical finding suggests that the bone marrow niche may negatively influence the recovery of normal and abnormal hematopoiesis after lethal irradiation.

### Collagen production in *MPL*^W515L^-transduced bone marrow MSCs

**Fig 3:**
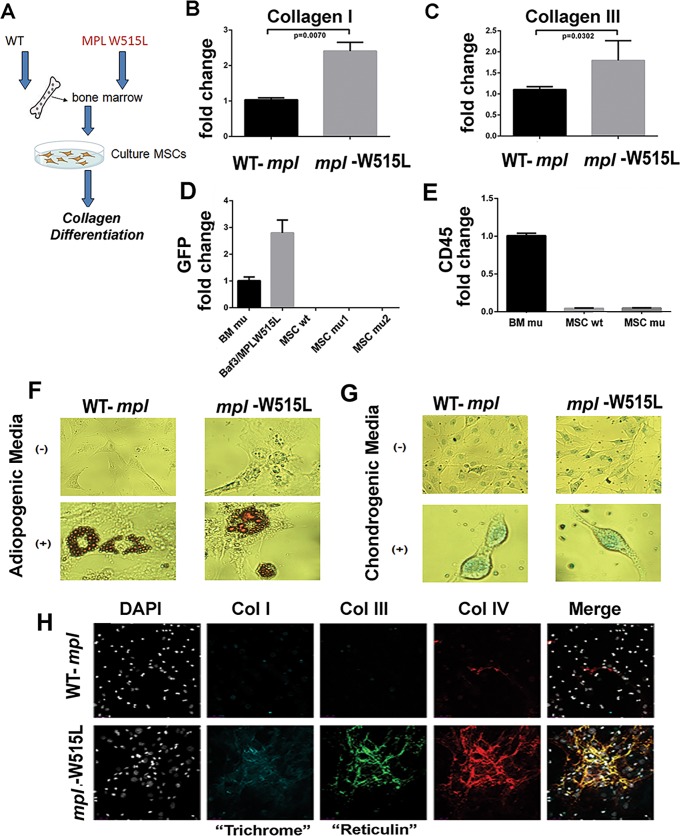
Analysis of MSCs. (A) Diagram of study methods for isolation of MSC populations. *MPL*^*WT*^ was used as a comparator to account for any possible influence of viral infection on the recovery or activity of the isolated MSCs. (B) mRNA of collagen I (*Col1A1)*, (C) mRNA for collagen III (*Col3A1*), (D, E) qRT-PCR for EGFP and CD45, respectively using hematopoietic cells expressing *MPL*^*W515L*^ (BM mu), BaF3 B-cell leukemia cell line overexpressing *MPL*^*W515L*^ (Baf3MPLW515L) as a positive control, MSCs from mice expressing *MPL*^WT^ (MSC wt) and MSCs derived from bone marrow of two mice expressing *MPL*^*W515L*^ (MSC mu1 and mu2). Cells exposed to no differentiation-inducing media and differentiation-inducing media specific for adipocytes and chondrocytes. Stain with Oil-red-O (F) or alcian blue (G) to detect adipogenic and chondrogenic populations. (H) Collagen I, III, and IV complexes stained after 72 hours of culture on glass slides. DAPI stained nuclei are shown in gray. Merged images shown are representative of 3–4 slides per test condition.

Both the production of cytokines and regulation of the extracellular matrix may be altered by the presence of mutant myeloid precursors. We found previously that cultured MSCs from fibrotic human bone marrow have the ability to undergo tri-lineage differentiation[13]. The lacked of CD45 (ie, CD45-) expression in cultured cell lines, and a cellular phenotype distinct from differentiated fibroblast[13], suggests that these cells meet the minimal criteria for mesenchymal stem cells. Cultured stromal populations from the bone marrow recreate three-dimension collagen fibers *in vitro*, which is evident by polychromatic immunofluorescence staining. Collagen I and III are clinically relevant since they are recognized by trichrome and reticulin on biopsy specimens, respectively[16]. We have also found that collagen IV and V are present in human patients with fibrosis. Staining for collagen VI, VIII, IX and XIII is negative in mouse MSC cultures (data not shown) under these experimental conditions. MSCs derived from *MPL*^W515L^ and *MPL*^*WT*^ (Fig 3A) expressing mice were compared for collagen production. In mice with MPN caused by *MPL*^*W515L*^, the MSC cultures expressed more abundant collagen I and III mRNA (Fig 3B and 3C, respectively). To ensure that changes within the mesenchymal compartment were unrelated to viral infection or presence of residual hematopoietic cells, qRT-PCR was performed to quantify EGFP (Fig 3D), CD45 (Fig 3E), and *MPL* (data not shown), respectively. All transcripts were undetectable in cultured MSCs after two passages, but were readily detectable in hematopoietic cells and in Baf3 cells transformed with *MPL*^*W515L*^ virus. Moreover, Oil-red-O positive adipocytes and alcian blue-positive chondrocytes were induced using adipogenic media (Fig 3F) and chondrogenic media (Fig 3G), respectively suggesting that the cells meet minimal criteria defining progenitor populations of MSCs[17]. Consistent with increased mRNA expression, excessive amounts of collagen I, III and IV were secreted into the extracellular matrix (Fig 3H) consistent with both increased production and polymerization of collagen fibers. Moreover, prolonged MSC cultures grown until the induction of senescence-associated cell death (roughly 90 days or five passages) continued to produce excessive collagen mRNA and to deposit greater amounts of collagen complexes (data not shown) in the absence of exogenous cytokines and hematopoietic cells.

### Stable reprogramming of MSCs derived from *MPL*^*W515L*^-transplanted mice

**Fig 4:**
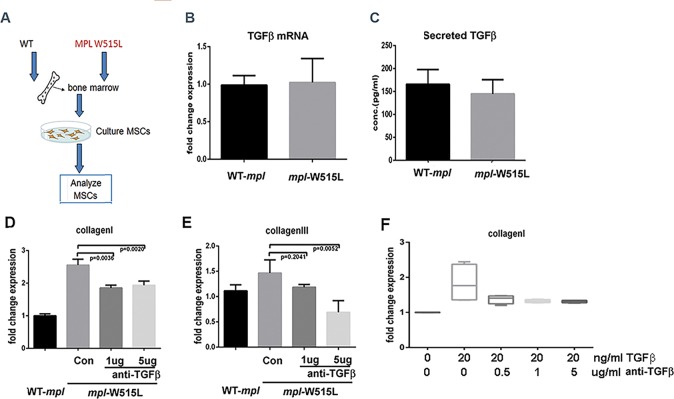
Production of TGFβ and anti-TGFβ in *MPL*^*WT*^ and *MPL*^W515L^-transduced bone marrow-derived MSC. (A) Diagram showing conditions used for this experiment. mRNA expression (B) and secreted levels (C) of TGFβ from MSCs derived from mice transplanted with bone marrow expressing *MPL*^*WT*^ or *MPL*^*W515L*^ retroviruses. Collagen I (*Col1A1*) (D) and III (*Col3A1*) (E) mRNA expressed in these cells in the presence of anti-TGFβ (1 and 5 μg/ml) monoclonal antibody during a three day culture period. (F) Collagen I (*Col1A1*) mRNA in MSCs derived from *MPL*^*W*T^ mice under treatment with TGFβ and anti-TGFβ antibody. Relative expression to TATA binding protein (TBP) control is shown. Statistical analysis was performed using 2-sided t test with significance <0.05. Experiments are shown for three biological replicates.

Using primary murine MSCs that were never exposed to transplantation, exogenous TGFβ increased collagen protein production and transcription as expected (data not shown). TGFβ from transformed megakaryocytes or neutrophils is hypothesized to modulate osteoblasts and/or mesenchymal stem cell populations to produce more collagen fibers [10, 11, 18, 19]. Our data in Fig 3 demonstrates that collagen excess is a stable feature of cells derived from the malignant microenvironment. Since the pro-fibrotic phenotype is evident in cultures in the absence of HPCs, we determined if *MPL*^W515L^ transplantation induces a TGFβ feed-forward autocrine loop associated with increased TGFβ production. Using primary MSCs isolated from bone marrow transplant recipients expressing *MPL*^*WT*^ or *MPL*^W515L^ (Fig 4A), the expression of TGFβ mRNA was measured by qRT-PCR relative to TBP mRNA (Fig 4B) and the total amount (both active and inactive) of TGFβ secreted into the culture supernatant (Fig 4C) was examined. TGFβ is present at approximately 150 pg/ml in both *MPL*^W515L^- and *MPL*^*WT*^ transplant-derived MSCs. To determine if TGFβ antibody can modulate the amount of collagen produced by these MSCs, collagen I and III was examined from bone marrow derived MSCs in the presence of neutralizing anti-TGFβ antibody. While there was a dose-dependent reduction in collagen III, collagen I repression was incomplete and not fully dose dependent comparing the 1 and 5 μg/ml dose (Fig 4D and 4E). Therefore, the efficacy of the antibody was confirmed by treating MSCs from *MPL*^*WT*^ transplanted mice with 20 ng/ml of recombinant TGFβ to induce Collagen I. A reduction by approximately 90% (Fig 4F) in collagen I occurred with doses of the antibody ≥ 0.5 μg/ml. These results show that the antibody can neutralize up to 20ng/ml of TGFβ from MSCs and suggests that the stable increase in collagen is at least partially independent of a feed-forward loop caused by increased autocrine TGFβ secretion.

### Altered signaling pathways in MSCs from *MPL*^W515L^-tranplanted mice

**Fig 5:**
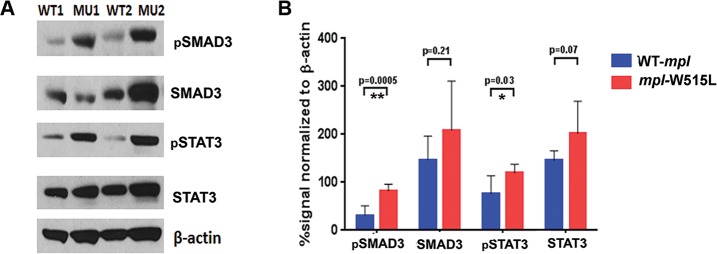
Elevated signaling pathways induced stably in MSCs from animals with MPN. (A) Western blot analysis with (B) quantification of the bands for pSmad3, Smad3, pSTAT3, STAT3, and β-actin. Shown are cell extracts from WT1 and WT2 cell lines (MSCs derived from bone marrow expressing *MPL*^*WT*^) and MU1 and MU2 (MSCs derived from bone marrow expressing *MPL*^*W515L*^). The experimental results are representative of three. Statistical analysis was performed using 2-sided t test with significance <0.05.

Emerging evidence suggests that MSCs are affected by the tumor and that the tumor may be reciprocally reshaped by the microenvironment. Signaling intermediates of the TGFβ superfamily are activated through ligand-induced phosphorylation. TGFβ and activin phosphorylate Smad2 and Smad3, whereas, bone morphogenetic protein (BMP) signaling leads to pSmad1, 5, and 9. Since MSCs from *MPL*^*WT*^ and *MPL*^*W515L*^ transplant recipients express comparable TGFβ, we investigated phosphorylated Smad3 (pSmad3) and pSTAT3; an alternative pathway associated with fibrosis through TNFα and IL-6 (Fig 5). pSmad3 was found to be significantly higher in MSCs derived from *MPL*^*W515L*^ transplanted mice. Furthermore, pSTAT3 was also significantly higher in this cell population. Total Smad3 and total STAT3 were not overexpressed suggesting that cellular reprogramming leads to alternative signaling events.

### FGFb reverses the fibrotic phenotype of *mpl*^W515L^- MSCs

**Fig 6:**
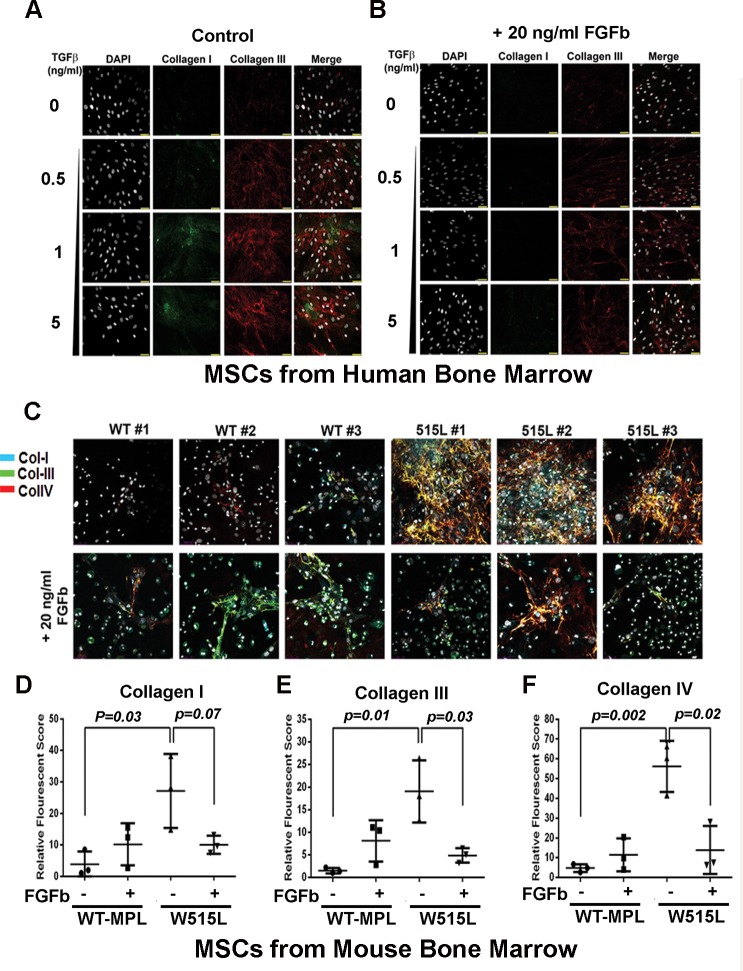
FGFb reverses the fibrotic phenotype of *MPL*^W515L^- MSCs. FGFb and LIF maintain MSCs in a pluripotent state, but stimulate proliferation and expansion. (A, B) MSCs derived from human bone marrow were exposed to increasing concentrations (0.5, 1, 5 ng/ml) of TGFβ to enhance collagen I (green) and collagen III (red) in three-dimensional culture conditions in the absence (A) and presence of 20 ng/ml FGFb (B). (C) MSCs from three different mice expressing *MPL*^*WT*^ or *MPL*^W515L^ which has increased expression of collagen I, III, and IV. Presence of 20 ng/ml FGFb reduces collagen deposition in the culture after 72 hours. Cell numbers were increased after continuous culture in this growth factor (data not shown). Quantification of collagen I (D) collagen III, (E) collagen IV (F) fibers under each condition shown with p-values indicated based on analysis using a 2-sided t-test.

Since MSCs can be used for cell-based therapies to reverse fibrosis [20–24], we examined the effect of FGFb on proliferation and on collagen production. Both FGFb [25] and leukemia inhibitory factor (LIF) [26] allow MSCs to maintain a multipotent phenotype while stimulating cell cycle progression (S3 Fig). Using TGFβ cultured human bone marrow-derived MSC and MSCs cultured from *MPL*^*WT*^ and *MPL*^W515L^-transduced bone marrow, we determined the effect of FGFb on collagen deposition using polychromatic immunofluorescence. As shown in Fig 6A, collagen I and III was evident in human MSCs after exposure to increasing doses of TGF-β, as expected. Co-culture of these cells with 20 ng/ml of FGFb for 72 hours significantly reduced collagen matrix deposition Fig 6B. Next, similar experiments were performed with *MPL*^WT^ and *MPL*^W515L^ MSCs. After 72 hours, cell cultures were stained to visualize complexes of collagen I, III and IV (Fig 6C). Cultures from three different mice were compared. We have previously demonstrated that the visual structural organization of collagen can be quantified and measured using three-dimensional images of deposited collagen fibers [13]. Analysis of collagen production by immunofluorescence showed a significant reduction in total collagen fibrils due to FGFb treatment when the mean pixel intensity value was calculated for the entire field (Fig 6D–6F).

## Conclusions

MSCs were isolated from mice with MPN induced through *MPL*^W515L^-transplantation [8] and collagen I, III and IV expression was significantly modified in continuously grown cultures ex vivo. In this model, the altered production and deposition of collagen persisted in the absence of malignant cells, as shown with CD45 qRT-PCR, suggesting that an intrinsic program is altered in the MSC population. Lack of GFP and *MPL* gene expression in the MSCs further confirms that the phenotype is independent of bystander or direct viral infection or mutant gene expression.

Fibrosis in MPN patients is thought to result from the production of environmental stress factors[10, 11, 18, 19, 27, 28] that are secreted by mutant hematopoietic cells. In a disease previously studied by our group (ie,LGL leukemia), mature CD8+ T-cells in the bone marrow are known to secrete a number of inflammatory cytokines[29, 30] that may be responsible for the induction of fibrosis. Neoplastic lymphocytes in this disease are characteristically present in the liver, spleen and bone marrow of patients that display symptomatic splenomegaly, anemia, neutropenia, and autoimmune phenomenon such as rheumatoid arthritis and serum abnormalities[29–33]. Our studies showed that bone marrow fibrosis severity is significantly correlated with the number of T-cell LGLs present in the bone marrow, and likewise the presence and severity of cytopenias[13]. In the *MPL*^*W515L*^-associated MPN mouse model, progressive remodeling of the endosteal bone marrow niche has been reported to occur via the presence of cytokines such as thrombopoietin, CCL3, TGFβ, and Notch signaling[34]. Unlike the growth promoting effect of TGFβ in peripheral tissue fibroblasts[35] such as stellate cells that induce fibrosis in the liver, TGFβ generally slows proliferation in bone marrow-derived MSCs[36] while increasing the production of collagen[37] [38–41]. Although TGFβ is one of the key factors linked to fibrosis in this disease[35, 42–44], MSCs exposed to blocking TGFβ antibodies only partially reduced the deposition of collagen in continuously cultured cells suggesting that other factors may be important including, 1) other TGF-beta family members, or 2) loss of suppressive signaling pathways. Increased phosphorylation of the transcription factor STAT3 and pSmad3 was detected in the *MPL*^*W515L*^-derived MSCs suggesting that suppressive signaling events may be diminished or that there are aberrant cooperative intracellular signaling networks activated. Many inflammatory cytokines, including IL-6, TNF and IL-1, activate the Ras/MAPK pathway to initiate a signaling circuits involving STAT3 [45]. Ras-activated STAT3 has been shown to synergize with TGF-β in other cell systems[46] so it is possible that increased intracellular signaling networks in response to a combination of factors may contribute to the continued production of collagen in the absence of malignant hematopoietic cells.

Thus far, it has been difficult to reverse the effects of excessive collagen deposition in MF and to restore altered extracellular matrix regulation in fibrotic bone marrow diseases. MPN clones persist after JAK2 inhibitor therapy partially due to altered utilization of JAK1 and TYK2 heterodimers [47]. Manshouri et al [48] showed that stromal cells co-cultured with *JAK2*^*V617F*^ expressing tumor cells provide a protective signal that attenuates JAK2 inhibitor therapy. Only stem cell transplantation has been shown to reverse fibrosis[49]. Our data showing cellular reprogramming of the MSCs is mechanistically consistent with this observation.

FGFb is known to antagonize TGFβ1 signaling [50]. Yoon et al showed previously that FGFb protein expression is reduced in bone marrow mesenchymal cells of patients with MPNs using computerized image analysis of immunohistochemical stains [51] while simultaneously increased in the malignant megakaryocytes of MPN patients. Interestingly collagen deposition from the cultured MSCs was almost completely reversed after exposure to FGFb. Normal MSCs exposed to FGFb maintain a pluripotent gene expression signature [13], have elongated telomeres, and display steadily increasing population doublings [25]. While there is no evidence suggesting that suboptimal FGFb levels contribute to the constitutive activation of Smad3/STAT3 or reprogramming, the response to exogenous FGFb is shows that the aberrant signaling circuit is reversible. Stem cell transplantation with MSCs has proven to be very effective at reducing fibrosis in other organs such as the kidney, lung and liver [20–24]. The clinical efficacy of JAK2 inhibitor therapy may be improved by revitalizing the bone marrow niche. In this regard, additional experiments with human MSCs are warranted.

Additional information is needed to determine the basis for elevated pSmad3 and pSTAT3 signaling as the targeted pharmacological disruption of these events may reverse the fibrotic phenotype. Our results demonstrate a similar mechanism of pathogenesis in the *MPL*^W515L^ mouse model as compared to patients with LGL leukemia suggesting that common mechanisms may underlie bone marrow fibrosis in these two hematological malignancies.

## Supporting Information

S1 Fig WHO classification of chronic myeloid neoplasms related to MPNs. (DOCX)

S2 Fig Overview of retroviral transduction methods. (DOCX)

S3 Fig Osteoblast differentiation. (DOCX)
