# Supplementary material for: Mesenchymal Cell Reprogramming in Experimental MPLW515L Mouse Model of Myelofibrosis
Source: PLoS One. 2017 Jan 30;12(1):e0166014. doi: 10.1371/journal.pone.0166014 (PMC5279751; doi:10.1371/journal.pone.0166014)
Supplement: S1 Fig — (DOCX) [file pone.0166014.s001.docx]

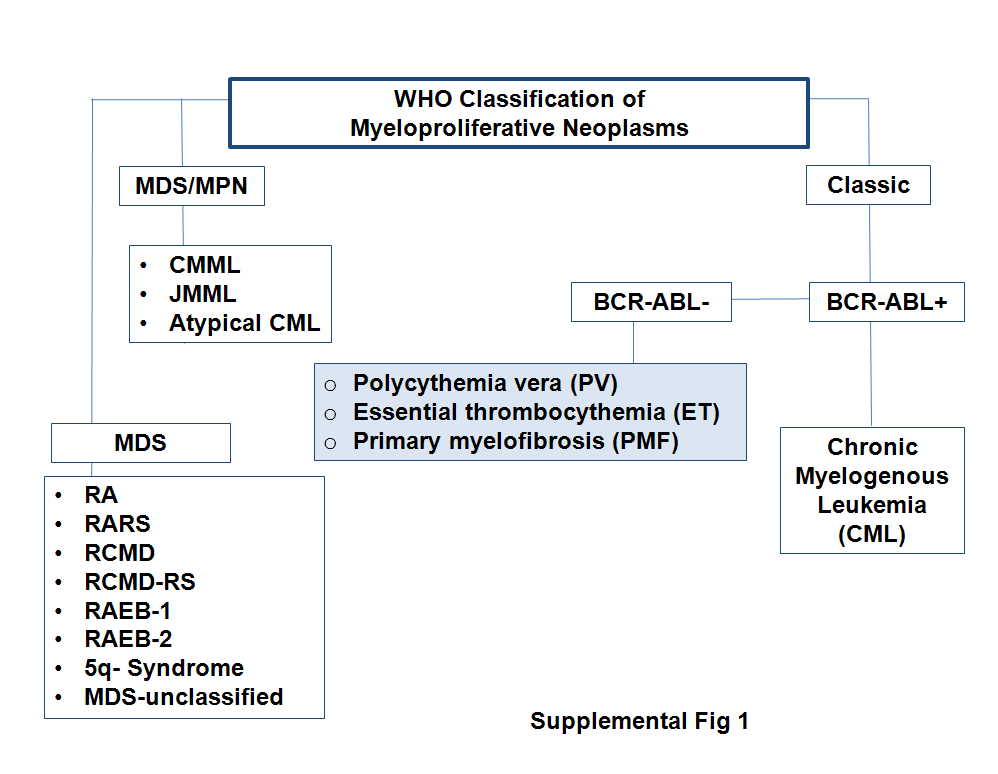


S1 Fig. WHO classification of chronic myeloid neoplasms related to MPNs. MDS=myelodysplastic syndrome, CMML=chronic myelomonocytic leukemia, JMML=Juvenile myelomoncytic leukemia, CML=chronic myelogenous leukemia, RA=refractory anemia, RCMD=refractory cytopenia with multilineage dysplasia, RS=ring sideroblast, RAEB=refractory anemia with excess blasts.
