# Supplementary material for: Mesenchymal Cell Reprogramming in Experimental MPLW515L Mouse Model of Myelofibrosis
Source: PLoS One. 2017 Jan 30;12(1):e0166014. doi: 10.1371/journal.pone.0166014 (PMC5279751; doi:10.1371/journal.pone.0166014)
Supplement: S2 Fig — (DOCX) [file pone.0166014.s002.docx]

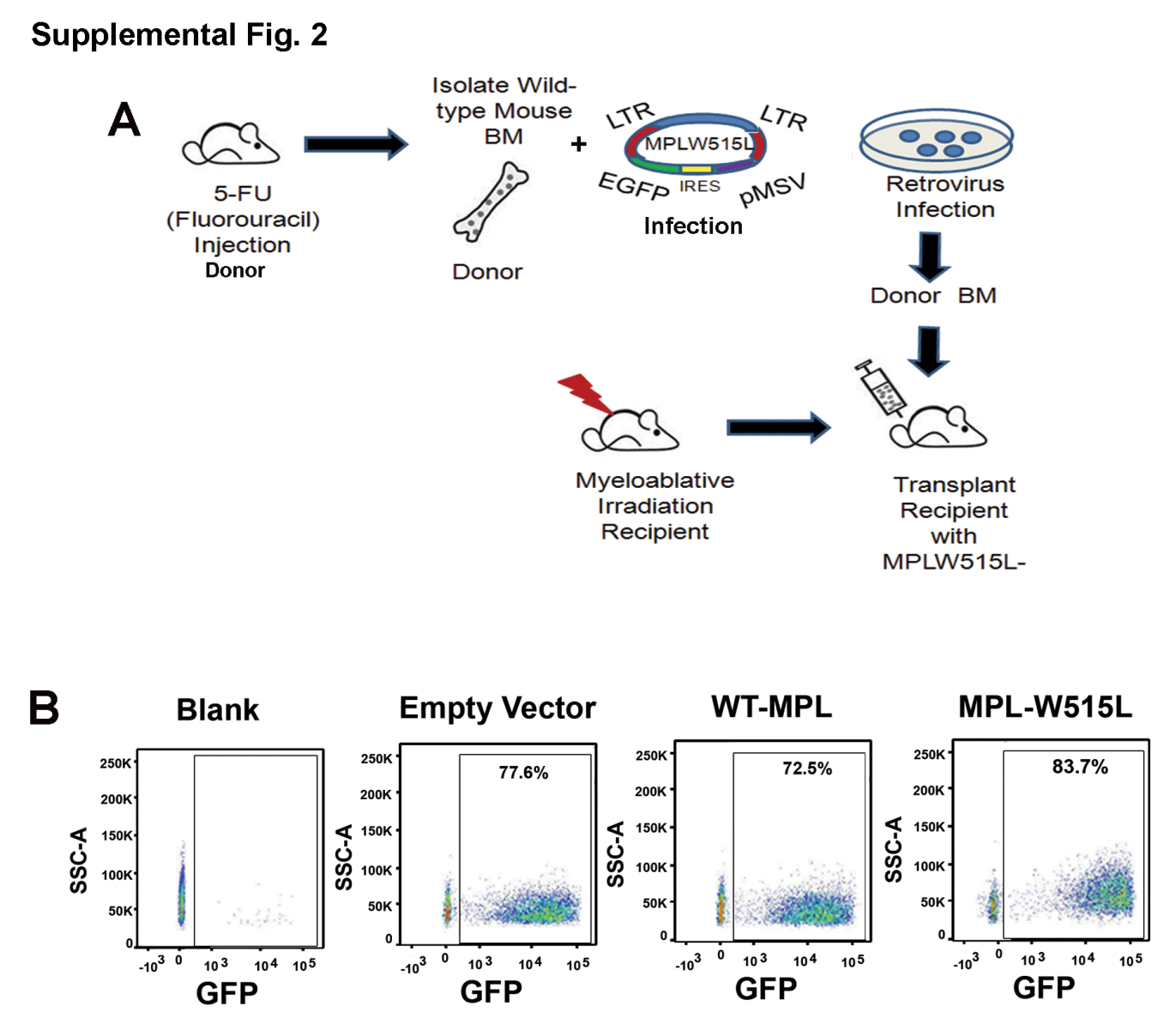
S2 Fig. Overview of retroviral transduction methods.

(A) Diagram showing MPN model induced by retroviral transduction of *mpl^W515L^*^.^. (B) Expression of GFP from each retroviral construct in NIH-3T3.
