# Supplementary material for: Mesenchymal Cell Reprogramming in Experimental MPLW515L Mouse Model of Myelofibrosis
Source: PLoS One. 2017 Jan 30;12(1):e0166014. doi: 10.1371/journal.pone.0166014 (PMC5279751; doi:10.1371/journal.pone.0166014)
Supplement: S3 Fig — (DOCX) [file pone.0166014.s003.docx]

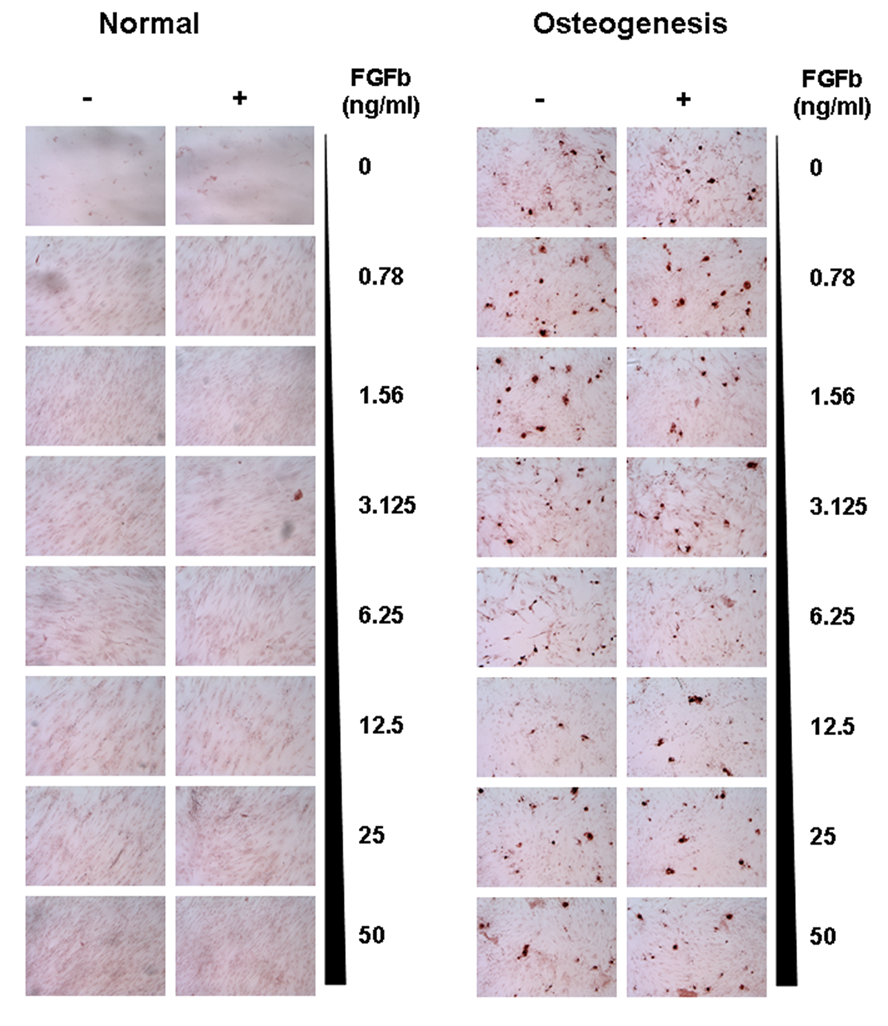


S3 Fig. Osteoblast differentiation.

Culture of human MSCs with non-differentiating (normal) media and osteoblast inducing media in the absence of FGFb and presence of increasing doses of FGFb (0.78, 1.56, 3.125. 6.25, 12.5, 25, 50 ng/ml. Cells were then stained with alizarin red S[[12](#_ENREF_12)].
